# The Impact of Addition of Consolidation Chemotherapy to Standard Cisplatin-Based Chemoradiotherapy in Uterine Cervical Cancer: Matter of Distant Relapse

**DOI:** 10.1155/2019/1217838

**Published:** 2019-03-11

**Authors:** Vanessa A. Fabri, Ana C. M. Queiroz, Henrique Mantoan, Solange M. Sanches, Andrea P. G. Guimarães, Adriana R. G. Ribeiro, Ronaldo P. Souza, Joyce M. L. Maya, Elizabeth S. Santos, Fabrício S. Castro, João P. N. S. Lima, Michael J. Chen, Glauco Baiocchi, Alexandre A. B. A. da Costa

**Affiliations:** ^1^Medical Oncology Department, A.C.Camargo Cancer Center, Sao Paulo, 211 Professor Antonio Prudente Street, 01509-900 Liberdade, Brazil; ^2^Gynecology Oncology Department, A.C.Camargo Cancer Center, Sao Paulo, 211 Professor Antonio Prudente Street, 01509-900 Liberdade, Brazil; ^3^Radiotherapy Department, A.C.Camargo Cancer Center, Sao Paulo, 211 Professor Antonio Prudente Street, 01509-900 Liberdade, Brazil

## Abstract

**Background:**

Treatment of advanced uterine cervical cancer has advanced little in the last 15 years. Although two phase III trials showed survival benefit with the addition of consolidation chemotherapy (CT) after cisplatin-based chemoradiotherapy (RTCT), it is not considered standard of care. We aimed to evaluate the benefit of consolidation CT compared to no additional treatment in patients treated with RTCT.

**Methods:**

This is a retrospective study including 186 patients with FIGO stage IB2, IIA2, or IIB to IVB (paraaortic lymph nodes only) uterine cervical cancer who were treated with standard RTCT alone or RTCT followed by consolidation CT. Overall survival (OS), progression free survival (PFS), and the risk of distant and local relapses were compared between the two treatment groups.

**Results:**

At 3 years OS was 91% versus 82.3% (p=0.027), PFS 84.3% versus 54.4% (p=0.047), and distant metastasis free survival (DMFS) 80.4% versus 62.5% (p=0.027) in favor of the consolidation CT group. Multivariate analysis confirmed the benefit of consolidation CT. There was no difference in locoregional free survival (LRFS). Positive lymph node was related to a higher risk of distant relapse. In the lymph node positive subgroup consolidation CT resulted in longer OS (p=0.050), PFS (p=0.014), and DMFS (p=0.022); in the lymph node negative subgroup there was no benefit from consolidation CT.

**Conclusions:**

Use of consolidation CT resulted in longer OS and PFS, mostly due to control of distant relapses. Patients at higher risk of distant relapse showed the greatest benefit. This data generates a hypothesis that could help to better select patients to consolidation CT.

## 1. Introduction

Uterine cervical cancer is one of the most prevalent neoplasms among women in the developing word. There were 569,847 new cases and 311,365 deaths due to uterine cervical cancer worldwide in 2018[1]. In Brazil there are 16,360 new cases expected in 2018, making it the third most frequent neoplasm among women. In the North Region of Brazil it is the first most frequent among women and in the Northeast and Middle-West it is the second most frequent among women [2].

Locally advanced disease (FIGO stages IIB to IVA) and bulky disease (FIGO stages IB2 and IIA2) are treated with definitive radiotherapy (RT) with concomitant chemotherapy (CT). Several trials proved the benefit of the addition of concomitant CT to RT [3–10], but the survival rates are still unsatisfactory especially for more advanced disease as shown by the latest of these trials that found an OS at 5 years of 54% for patients with FIGO stage IIIB [10].

In the last 7 years the use of systemic CT beyond the concomitant phase of CT and RT (RTCT) has been studied as a treatment intensification strategy. Initial studies with induction CT prior to RTCT have not shown promising results leading to questions about the novel drug combinations and timing of therapy [11, 12]. Two phase III trials have shown an OS benefit with the addition of consolidation CT after concomitant RTCT [13, 14]. Dueña-Gonzalez at al. evaluated the addition of two cycles with gemcitabine and cisplatin after a concomitant phase with gemcitabine and cisplatin while Tang et al. evaluated the addition of one cycle with cisplatin and paclitaxel prior to RTCT and two cycles after RTCT [14]. The benefit was mostly due to distant relapse control. Despite the OS benefit showed in the two trials, consolidation CT is not considered a standard of care[15, 16], mostly due to statistical flaws and the excessive toxicity of the experimental treatment used in the first study [13] and the inclusion of only patients diagnosed with adenocarcinoma histology in the second study [14].

In this retrospective study we aimed to evaluate the benefit of consolidation CT compared to no additional systemic treatment in uterine cervical cancer patients treated with RTCT.

## 2. Material and Methods

### 2.1. Patients

From 2005 to 2017, 227 patients with FIGO stage IB2, IIA2, or IIB to IVA and IVB paraaortic lymph nodes only) uterine cervical cancer were treated with definitive RT at A.C.Camargo Cancer Center, São Paulo, Brazil. Twenty-eight patients did not receive concomitant CT and 13 patients had no data regarding the use of consolidation CT after RTCT and were excluded, leading to 186 patients included in the present study. Patients with rare histological types (neuroendocrine tumors and sarcomas) were not included. A retrospective review of the medical records was performed. The Ethics Committee of the A.C.Camargo Cancer Center approved this study (CEP# 2219/16).

### 2.2. Clinical Data and Endpoints

Clinical findings were retrieved from the medical records including age, ECOG performance status at the beginning of treatment, histology, histological grade, tumor size, lymph node status, dose of radiotherapy planned, method of RT used (3D versus 2D), type of CT used in association with RT in the concomitant, number of cycles of concomitant CT, use of consolidation CT, type of CT used as consolidation CT, number of cycles of consolidation CT, date of disease progression, sites of disease recurrence, treatments after progression, and date of death or last follow-up. We adopted the FIGO stage as identified at gynecological examination. All patients had data on abdomen and pelvic computer tomography or magnetic resonance image to evaluate lymph node status. No patient received surgical staging of the paraaortic lymph nodes.

The endpoints analyzed were overall survival (OS) and progression free survival (PFS). Distant metastasis (DMFS) and locoregional recurrence free survival (LRFS) were also evaluated. Locoregional relapse was defined as recurrence or progression within the pelvis as reported by computer tomography or magnetic resonance image reports. Distant relapse was defined as recurrence outside pelvis as reported by computer tomography, magnetic resonance image or bone scans reports. OS was calculated from diagnosis until death by any cause. PFS was calculated from diagnosis until progression or death by any cause. DMFS and LRFS were calculated from diagnosis until the identification of distant metastasis and locoregional relapse, respectively.

### 2.3. Treatment

At A.C.Camargo Cancer Center the standard treatment for patients with locally advanced disease is with external pelvic RT given as a 1.8 Gy fraction daily, five days per week up to the total dose of 50.4Gy. After external RT, patients received high dose rate brachytherapy in 4 fractions of 7.0 Gy to a total dose of 80Gy to the point A. Patients with positive lymph nodes on imaging (or biopsy proven) receive additional local irradiation (“boost”) up to 54 Gy. For paraaortic sites, locorregional drainage is treated up to 45 Gy, concomitantly to pelvic irradiation and positive nodes receive additional boost up to 50-54 Gy, at physician discretion. Cisplatin at a dose of 40mg/m^2^ is infused weekly during RT for a minimum of 5 cycles. Consolidation CT with two cycles of cisplatin 50mg/m^2^ D1 and gemcitabine 1000mg/m^2^ D1 and 8 every 21 days for 2 cycles is an option according to the treating physician discretion.

### 2.4. Statistical Analysis

Statistical analyzes were performed using the SPSS (v. 23.0; SPSS, Chicago, IL, USA) software, adopting a two-tailed P< 0.05 value as significant. Chi-square or Fisher's Exact test were used for comparison of categorical data. Survival curves for OS, PFS, LRFS, and DMFS were constructed using the Kaplan–Meier method and compared using the Log-Rank Test. A Cox proportional hazards regression model was used for multivariate analysis; all variables with p < 0.05 in the univariate analyses were included in the multivariate analyses. After identification of risk factors for shorter DMFS we evaluated the impact of consolidation CT in subgroups according to the category of the variables associated with DMFS. A propensity score analysis was done. We calculated the propensity score using a logistic regression model including characteristics that were unbalanced between the two treatment groups. A multivariable model for OS and PFS was done including the propensity score and the treatment group. Moreover, we did a sensitive analysis in a propensity score matched cohort for OS and PFS.

## 3. Results

One hundred and eighty-six patients were included. Median age was 48.0 years-old (IQR 37.6-58.7), ECOG performance status was zero in 63.9% of patients, only two patients presented ECOG performance status of 2, tumor histology was squamous cell carcinoma in 76.4%, tumor histological grade was 2 in 58.2% and 3 in 34.0% of patients, FIGO stage IB2-IIA2 in 9.3%, IIB in 48.1%, and III-IV in 42.6% of patients, median tumor size was 5.0cm (IQR 41.0-60.0), and positive lymph nodes were present in 54.8% of patients. Clinical and pathological characteristics are summarized in Table 1.

Median follow-up time was 37.7 months. Forty-five deaths have occurred, 63 patients are experiencing disease progression, 52 have presented distant recurrences, and 23 have presented locorregional recurrences. Six of the 45 deaths were not caused directly by the cancer: 2 patients with FIGO stage IIB, 3 patients with FIGO stage IIIB, and 1 patient with positive paraaortic lymph node. For the whole cohort, at 3 and 5 years, OS was 74.3% and 69.5%, PFS was 60.9% and 56.6%, DMFS was 68.2% and 62.1%, and LRFS was 83.6% and 83.6%, respectively (Supplementary Figure S1).

### 3.1. Consolidation Chemotherapy versus Observation after RTCT

Fifty-eight patients were treated with consolidation CT. Only five patients (8.6%) could not receive the two cycles due to toxicity, while 53 (91.4%) received the 2 planned cycles.

Median follow-up times were 30.9 months for patients in the consolidation CT group and 45.5 months in the RTCT alone group (p<0.001).

**Figure 1:**
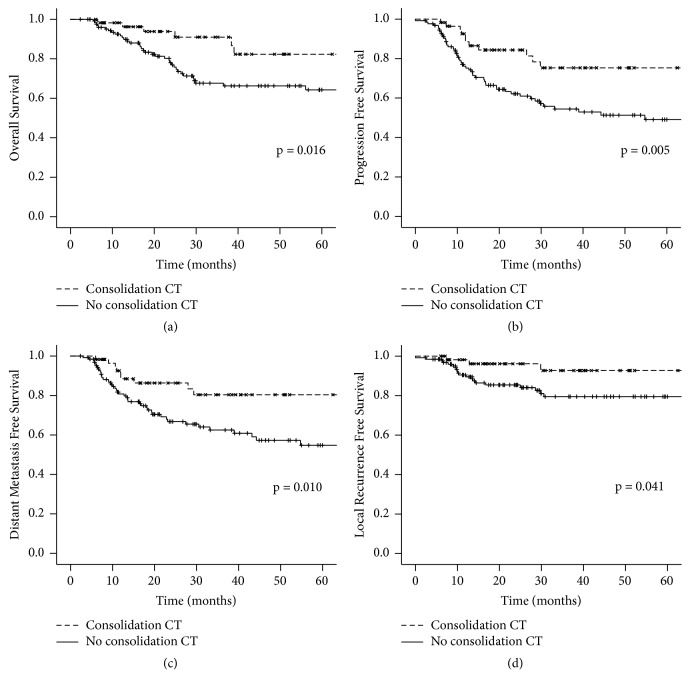
Survival outcomes according to groups by treatment with consolidation CT and without consolidation CT. (a) Overall survival; (b) progression free survival; (c) distant metastasis free survival; (d) locorregional free survival. CT = chemotherapy. All p values calculated by Log-Rank Test.

OS at 3 years was 91% versus 82.3% and at 5 years it was 82.3% versus 64.3% in favor of patients who were treated with consolidation CT. Median OS was not reached in both groups (p=0.016) (Figure 1). In the multivariate analysis the use of consolidation CT remained independently associated with a higher OS, with a decrease in the risk of death of 59% (HR 0.41; 0.16-0.99, 95%CI; p=0.047) (Table 2).

PFS at 3 years was 84.3% versus 54.4% and at 5 years it was 84.3% versus 49.1% in favor of patients who were treated with consolidation CT. Median PFS was not reached in the consolidation CT group and 54.8 months for patients who did not receive consolidation CT (p=0.027) (Figure 1). In the multivariate analysis the use of consolidation CT decreased the risk of progression by 61% (HR 0.39; 0.19-0.82, 95%CI; p=0.012) (Table 2).

DMFS at 3 years was 80.4% versus 62.5% and at 5 years it was 80.4% versus 54.8% in favor of patients who were treated with consolidation CT. Median DMFS was not reached in both groups (p=0.027) (Figure 1). In the multivariate analysis the use of consolidation CT remained independently associated with a higher DMFS (HR 0.43; 0.21-0.91, 95% CI, p = 0.028) (Table 3).

LRFS at 3 years was 92.7% versus 79.5% and at 5 years it was 92.7% versus 79.5% in favor of patients who were treated with consolidation CT. Median LRFS was not reached in both groups (p=0.027) (Figure 1). In the multivariate analysis the use of consolidation CT decreased the risk of progression by 41% but it was not statistically significant (HR 0.59; 0.26-1.32, 95%CI; p=0.199) (Table 3).

We did a sensitivity analysis excluding the 18 patients who did not receive brachytherapy. After excluding these patients, in the multivariate models, consolidation CT remained associated with OS (HR 0.41; 0.17-0.99, 95%CI; p = 0.047), PFS (HR 0.39; 0.19-0.82, 95%CI; p = 0.012), and DMFS (HR 0.40; 0.20-0.83, 95%CI; p = 0.0413).

### 3.2. Propensity Score Analysis

Age, number of concomitant cisplatin to RT cycles, and type of RT were unbalanced between the two treatment groups and were included in the propensity score. A multivariable model including the propensity score and the use of consolidation CT for OS and PFS showed a nonstatistically significant benefit of consolidation CT for OS and confirmed a significant benefit for PFS. For OS HR was 0.49 (0.20-1.21, 95% CI; p=0.124) and for PFS HR was 0.50 (0.25-0.98, 95% CI; p=0.043).

We then evaluated a selected sample of patients matching 58 patients treated with consolidation CT and 58 patients treated with RTCT alone one-to-one according to propensity score. Clinical characteristics were balanced between the two treatment groups (Supplementary Table S1). In the matched cohort OS at 3 years was 85.8% and PFS at 3 years was 69.3%. At 3 years OS was 91.0% versus 80.8% (p=0.364) and PFS was 75.3% versus 63.6% (p=0.113) in favor of consolidation CT group; however it was not statistically significant (Supplementary Figure S2).

### 3.3. Risk Factors for Death, Recurrence, Distant Recurrence, and Locorregional Recurrence

Histological grade 3 and FIGO stage ≥ IIIA were related to a shorter OS in univariate cox regression analysis (Supplementary Table S2 and Supplementary Figure S3). In the multivariate analysis FIGO stage ≥ IIIA remained independently associated with a shorter OS (HR 1.97; 1.00-3.85, 95% CI, p = 0.049) (Table 2).

Histological grade 3, positive lymph node, and concurrent CT < 6 cycles were related to shorter PFS in univariate analysis (Supplementary Table S3 and Supplementary Figure S3). In the multivariate analysis positive lymph nodes remained independently associated with a lower PFS (HR 2.48; 1.30-4.78, 95% CI, p = 0.006) (Table 2).

Positive lymph nodes and 2D conformal radiation therapy (RT2D) were related to shorter DMFS (Supplementary Table S4 and Supplementary Figure S4). In the multivariate analysis positive lymph node remained independently associated with a shorter DMFS (HR 2.99; 1.54-5.81, 95% CI, p = 0.001) (Table 3).

Histological grade 3, concurrent CT < 6 cycles, and FIGO stage ≥ IIIA were related to shorter LRFS (Supplementary Table S5 and Supplementary Figure S4). In the multivariate analysis these 3 variables remained independently associated with a shorter LRFS (Table 3).

### 3.4. Benefit of Consolidation Chemotherapy by Subgroups according to Risk of Distant Relapse

**Figure 2:**
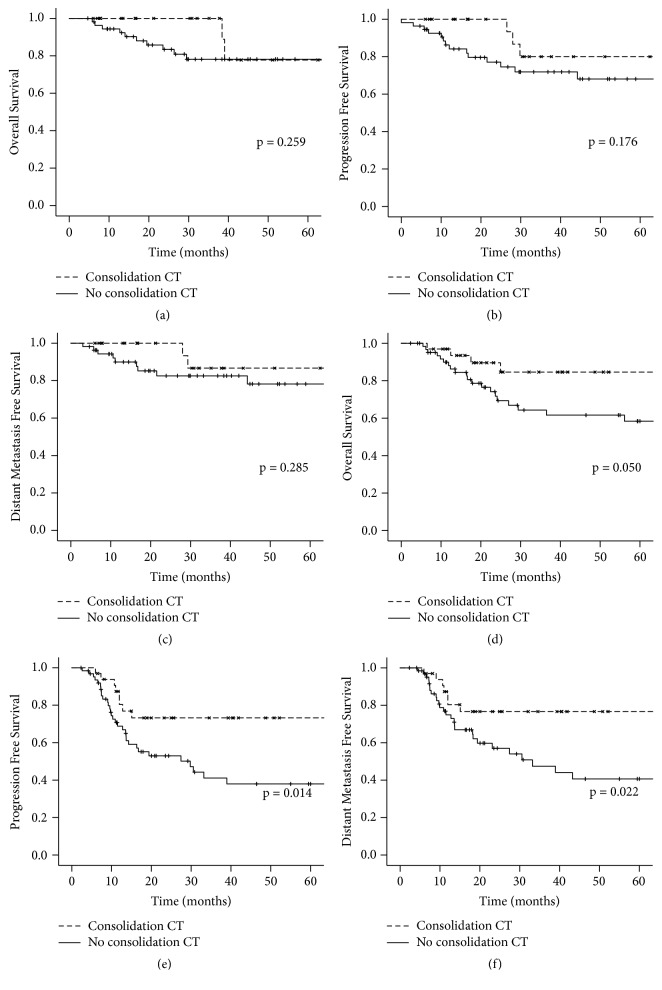
(a) Overall survival in node negative subgroup according to treatment; (b) progression free survival in node negative subgroup according to treatment; (c) distant metastasis free survival in node negative subgroup according to treatment; (d) overall survival in node positive subgroup according to treatment; (e) progression free survival in node positive subgroup according to treatment; (f) distant metastasis free survival in node positive subgroup according to treatment. CT = chemotherapy. All p values calculated by Log-Rank Test.

Positive lymph node was the risk factor with a stronger association with distant relapse. Once the benefit of consolidation CT was mostly due to control of distant relapse we tested if the benefit of consolidation CT was different in patients with negative lymph nodes and in patients with positive lymph nodes. In the node negative subgroup at five years, OS was 77.8% versus 78.2% (p=0.259), PFS was 80.0% versus 68.1% (p=0.176), and DMFS was 86.7% versus 78.2% (p=0.285), for consolidation CT and no consolidation CT, respectively. In the node positive subgroup, at five years, OS was 84.6% versus 58.4% (p=0.050), PFS was 73.2% versus 37.9% (p=0.014), and DMFS was 76.6% versus 40.7% (p=0.022), for consolidation CT and no consolidation CT, respectively (Figure 2). The interaction test between consolidation CT and lymph node status showed a p value of 0.227 for OS, 0.266 for PFS, and 0.419 for DMFS.

## 4. Discussion

In the present study we evaluated the role of consolidation CT in the treatment of 186 patients with uterine cervical cancer treated with definitive concomitant RTCT plus consolidation CT or RTCT alone. We found OS and PFS benefit for patients treated with consolidation CT mostly due to control of distant relapse. Lymph node status was associated with DMFS. When analyzing the impact of consolidation CT according to lymph node status the OS, PFS, and DMFS benefit of consolidation CT was seen only in the lymph node positive subgroup.

At least 3 other comparative studies support the benefit of consolidation CT. The Mexican phase III trial [13] evaluated the impact of two cycles of gemcitabine plus cisplatin as consolidation CT and found a 9% absolute improvement in the primary outcome of PFS at 3 years, from 65% to 74%. The experimental arm differed from the standard treatment also by the addition of gemcitabine to cisplatin in the concomitant phase hampering the conclusion of which of the two treatment intensification strategies were responsible for the survival improvement. The second study is a Chinese trial [14] that differed from ours by its prospective design, including only adenocarcinoma and using cisplatin plus paclitaxel as systemic chemotherapy, with one cycle prior to RT and two cycles after RT. PFS at 5 years was 71.4% versus 60.4% in favor of the systemic CT arm. The third study [17] is a Korean retrospective study including 80 patients treated with cisplatin plus 5FU concomitantly to RT and half of the patients treated with additional 3 cycle of cisplatin plus 5FU, PFS at 3 years of 74.4% versus 59.0% in favor of consolidation CT. Our study is the only study that included both squamous cell carcinoma and adenocarcinoma patients and treated patients with the standard concomitant treatment with weekly cisplatin and RT in both groups.

We found a benefit in DMFS in favor of consolidation CT and did not find a benefit of consolidation CT in LRFS. This is in accordance with the Mexican trial and the Korean study [13, 17], while the Chinese trial showed a benefit both in reduction of distant and local relapses[14].

In our study LRFS at 3 years was 92.7% versus 79.5% but the difference was not statistically significant in multivariate analysis. In the Mexican study local recurrences occurred in 16.4% of patients in the consolidation CT arm versus 11.2% in the RTCT alone arm and in the Korean study 10.3% versus 5.1%. In both studies these differences were not statistically significant. The lack of statistical significance may be due to the smaller frequency of local relapses compared to distant relapses in locally advanced cervical cancer treated with concomitant RTCT and to a weaker association of consolidation CT with reduction of local relapse making a large number of patients to prove a statistically significant difference necessary. Indeed, the Chinese study included 880 patients while the Mexican study included 515 patients. Thus the benefit of consolidation CT seems to be driven mostly by the control of distant recurrences but a benefit in local control can not be excluded.

Positive lymph nodes have been shown to be the most important prognostic factor for distant relapses in locally advanced cervical cancer [18–20]. We found a three times higher risk of distant relapse for patients with positive lymph nodes. Considering the hypothesis that the befit of consolidation CT is mostly driven by improvement of distant control we performed a subgroup analysis according to lymph node status. We found a benefit for consolidation CT regarding OS, PFS, and DMFS only in the subgroup of patients with positive lymph nodes.

The Mexican trial was the only other study that did a subgroup analysis [21]. They found a greater benefit of consolidation CT in patients with FIGO stage III and IV compared to stage IIB, adenocarcinoma compared to squamous cell carcinoma, in tumors larger than 5cm and in patients with positive paraaortic lymph nodes. There was no subgroup analysis according to pelvic lymph node status.

Our study confirmed the higher rate of distant relapse in lymph node positive patients and corroborates the hypothesis of greater benefit from consolidation CT in patients with higher risk of distant relapse. This finding could help to better identify patients for whom it is worthy to pursue with consolidation CT.

Our study has limitations intrinsic to its retrospective nature. Selection bias is an issue as we can see by the imbalance in baseline characteristics between the two groups. Patients in the consolidation CT were younger and completed 6 cycles of concomitant weekly cisplatin more often than the RTCT alone group. To address the selection bias we performed a Cox regression multivariable analysis including all variables related to each endpoint in the univariate analysis. The benefit of consolidation CT was confirmed in multivariate analysis for OS, PFS, and DMFS. Moreover, we performed a propensity score analysis doing a covariate adjustment using the propensity score and a sensitive survival analysis in a selected sample matched by the propensity score. In these two analyses the benefit of consolidation CT was not statistically significant anymore. This could be to a loss of statistical power in the propensity score analysis or due to a real confounding effect of the imbalance between the two treatment groups regarding age, number of concomitant cisplatin cycles, and use of 2D RT. Notably, age and 2D RT compared to 3D RT were not independently associated with any of the outcomes, and use of less than 6 cycles of concomitant CT was associated only with higher risk of local relapse. The lack of association of these variables with the outcomes points against their role as confounding factors even if it cannot be excluded. Moreover, there were less death events than progression events as expected, and consolidation CT remained associated with PFS but not with OS with p value less than 0.05. These findings put together suggest the higher p values in the propensity score analysis may be due more to lack of statistical power than to confounding effects. Median follow-up time is significantly shorter in the consolidation CT group, this implies more immature data on outcomes in this group, and longer follow-up is needed to confirm our initial results.

One strength of our study is that the two treatment groups differed in their treatment only in the use of consolidation CT, since all patients were treated with weekly cisplatin concomitantly to RT. Moreover, we have detailed data on sites of recurrences allowing the discrimination of factors related to distant and local relapses.

## 5. Conclusion

In conclusion the present study shows a benefit in OS and PFS of consolidation CT after RTCT compared to RTCT alone in uterine cervical cancer patients with bulky or locally advanced disease. Patients with higher risk of distant relapse such as those who present positive lymph node are those who mostly benefit from this treatment intensification strategy. We eagerly await the results from the phase III OUTBACK trial comparing 4 cycles of carboplatin plus paclitaxel after RTCT in patients with stage IB to IVA uterine cervical cancer.

## Figures and Tables

**Table 1:** Clinical characteristics of the patients.

Characteristic	Freq. (%)		
	Consolidation CT	No Consolidation CT	p*∗*
Number of patients	58 (31.2)	128 (68.8)	
Age (median/IQR)	41.5 (34.5 – 54.5)	51.3 (41.7 – 63.0)	< 0.0001
ECOG performance status			
0	36 (62.1)	81 (64.8)	0.720
≥ 1	22 (37.9)	44 (35.2)	
Histology			
Squamous cell carcinoma	41 (70.7)	98 (79.0)	0.217
Adenocarcinoma	17 (29.3)	26 (21.0)	
Grade			
1 and 2	34 (77.3)	59 (60.8)	0.056
3	10 (22.7)	38 (39.2)	
FIGO stage			0.637
IB2	3 (5.2)	7 (5.5)	
IIA2	2 (3.4)	5 (3.9)	
IIB	26 (44.8)	62 (48.8)	
IIIA	2 (3.4)	4 (3.1)	
IIIB	15 (25.9)	36 (28.3)	
IVA	9 (15.5)	7 (5.5)	
IVB^a^	1 (1.7)	5 (3.9)	

Tumor size			
< 6cm	28 (75.7)	56 (76.7)	0.904
≥ 6cm	9 (24.3)	17 (23.3)	
Lymph node			
Negative	25 (43.1)	55 (46.2)	0.732
Pelvic	32 (55.2)	60 (50.4)	
Paraaortic	1 (1.7)	4 (3.4)	
Concurrent CT < 6 cycles			
No	46 (79.3)	75 (64.7)	0.048
Yes	12 (20.7)	41 (35.3)	
Radiotherapy technique			
3D	53 (91.4)	88 (71.0)	0.002
2D	5 (8.6)	36 (29.0)	
Brachytherapy			
No	3 (5.2)	15 (12.0)	0.149
Yes	55 (94.8)	110 (88.0)	

*∗*All p values calculated using Chi square test or Fisher's exact test. CT = chemotherapy. IQR = *Interquartile* Range.

^a^ Paraaortic lymph nodes only.

**Table 2:** Multivariate analysis for overall survival and progression free survival.

	OS		PFS	
Characteristic	HR (95% CI)	p value	HR (95% CI)	p value
Consolidation CT				
No	1	0.047	1	0.012
Yes	0.41 (0.16-0.99)		0.39 (0.18-0.81)	
FIGO Stage				
< IIB	1	0.049	-	-
> IIIA	1.97 (1.00-3.85)		-	
Grade				
1 and 2	1	0.090	1	0.077
3	1.78 (0.91-3.47)		1.71 (0.94-3.10)	
Lymph node				
Negative	-	-	1	0.006
Positive	-		2.48 (1.29-4.75)	
Concurrent CT < 6 cycles				
No	-	-	1	0.071
Yes	-		1.78 (0.95-3.33)	

^1^136 patients with complete data included in the multivariate analysis, 35 events.

^2^ 135 patients with complete data included in the multivariate analysis, 46 events.

DMFS = distant metastasis free survival; LRFS = locorregional free survival; CT = chemotherapy.

**Table 3:** Multivariate analysis for distant metastasis free survival and locorregional free survival.

	DMFS^1^		LRFS^2^	
Characteristic	HR (95% CI)	p value	HR (95% CI)	p value
Consolidation CT				
No	1	0.028	-	-
Yes	0.43 (0.21-0.91)		-	
FIGO Stage				
< IIB	-	-	1	0.009
> IIIA	-		3.62 (1.37-9.49)	
Grade				
1 and 2	-	-	1	0.009
3	-		3.36 (1.35-8.32)	
Lymph node				
Negative	1	0.001	-	-
Positive	2.99 (1.54-5.81)		-	
Concurrent CT < 6 cycles				
No	-	-	1	0.022
Yes	-		2.82 (1.16-6.81)	
Radiotherapy technique				
3D	1	0.196		
2D	1.51 (0.81-2.80)			

^1^  174 patients with complete data included in the multivariate analysis, 46 events.

^2^ 133 patients with complete data included in the multivariate analysis, 20 events.

DMFS = distant metastasis free survival; LRFS = locorregional free survival; CT = chemotherapy.

## Data Availability

The data used to support the findings of this study are available from the corresponding author upon request.

## Supplementary Materials

Supplementary Materials Supplementary Figure S1: overall survival (A), progression free survival (B), distant metastasis free survival (C), and locorregional free survival (D) in the 186 patients treated with concomitant chemotherapy and radiotherapy. Supplementary Figure S2: survival outcomes according to groups by treatment with consolidation CT and without consolidation CT in the matched sample of 116 patients: (A) overall survival; (B) progression free survival. All p values calculated by Log-Rank Test. Supplementary Figure S3: (A) overall survival according to histological grade; (B) overall survival according to FIGO stage; (C) progression free survival according to histological grade; (D) progression free survival according to lymph node status; (E) progression free survival according to number of chemotherapy cycles concomitantly to radiotherapy. CT = chemotherapy. All p values calculated by Log-Rank Test. Supplementary Figure S4: (A) distant metastasis free survival according to lymph node status; (B) distant metastasis free survival according to radiotherapy technique; (C) locorregional free survival according to histological grade; (D) locorregional free survival according to number of chemotherapy cycles concomitantly to radiotherapy; (E) locorregional free survival according to FIGO stage. CT = chemotherapy. All p values calculated by Log-Rank Test. Supplementary Table S1: clinical characteristics of the one-to-one matched sample according to propensity. Supplementary Table S2: univariate analysis for overall survival. Supplementary Table S3: univariate analysis for progression free survival. Supplementary Table S4: univariate analysis for distance metastasis free survival. Supplementary Table S5: univariate analysis for locorregional free survival.
