# Supplementary material for: The Impact of Addition of Consolidation Chemotherapy to Standard Cisplatin-Based Chemoradiotherapy in Uterine Cervical Cancer: Matter of Distant Relapse
Source: J Oncol. 2019 Mar 11;2019:1217838. doi: 10.1155/2019/1217838 (PMC6432701; doi:10.1155/2019/1217838)
Supplement: Supplementary Materials — Supplementary Figure S1: overall survival (A), progression free survival (B), distant metastasis free survival (C), and locorregional free survival (D) in the 186 patients treated with concomitant chemotherapy and radiotherapy. Supplementary Figure S2: survival outcomes according to groups by treatment with consolidation CT and without consolidation CT in the matched sample of 116 patients: (A) overall survival; (B) progression free survival. All p values calculated by Log-Rank Test. Supplementary Figure S3: (A) overall survival according to histological grade; (B) overall survival according to FIGO stage; (C) progression free survival according to histological grade; (D) progression free survival according to lymph node status; (E) progression free survival according to number of chemotherapy cycles concomitantly to radiotherapy. CT = chemotherapy. All p values calculated by Log-Rank Test. Supplementary Figure S4: (A) distant metastasis free survival according to lymph node status; (B) distant metastasis free survival according to radiotherapy technique; (C) locorregional free survival according to histological grade; (D) locorregional free survival according to number of chemotherapy cycles concomitantly to radiotherapy; (E) locorregional free survival according to FIGO stage. CT = chemotherapy. All p values calculated by Log-Rank Test. Supplementary Table S1: clinical characteristics of the one-to-one matched sample according to propensity. Supplementary Table S2: univariate analysis for overall survival. Supplementary Table S3: univariate analysis for progression free survival. Supplementary Table S4: univariate analysis for distance metastasis free survival. Supplementary Table S5: univariate analysis for locorregional free survival. [file 1217838.f1.docx]

**Supplementary material:**

**
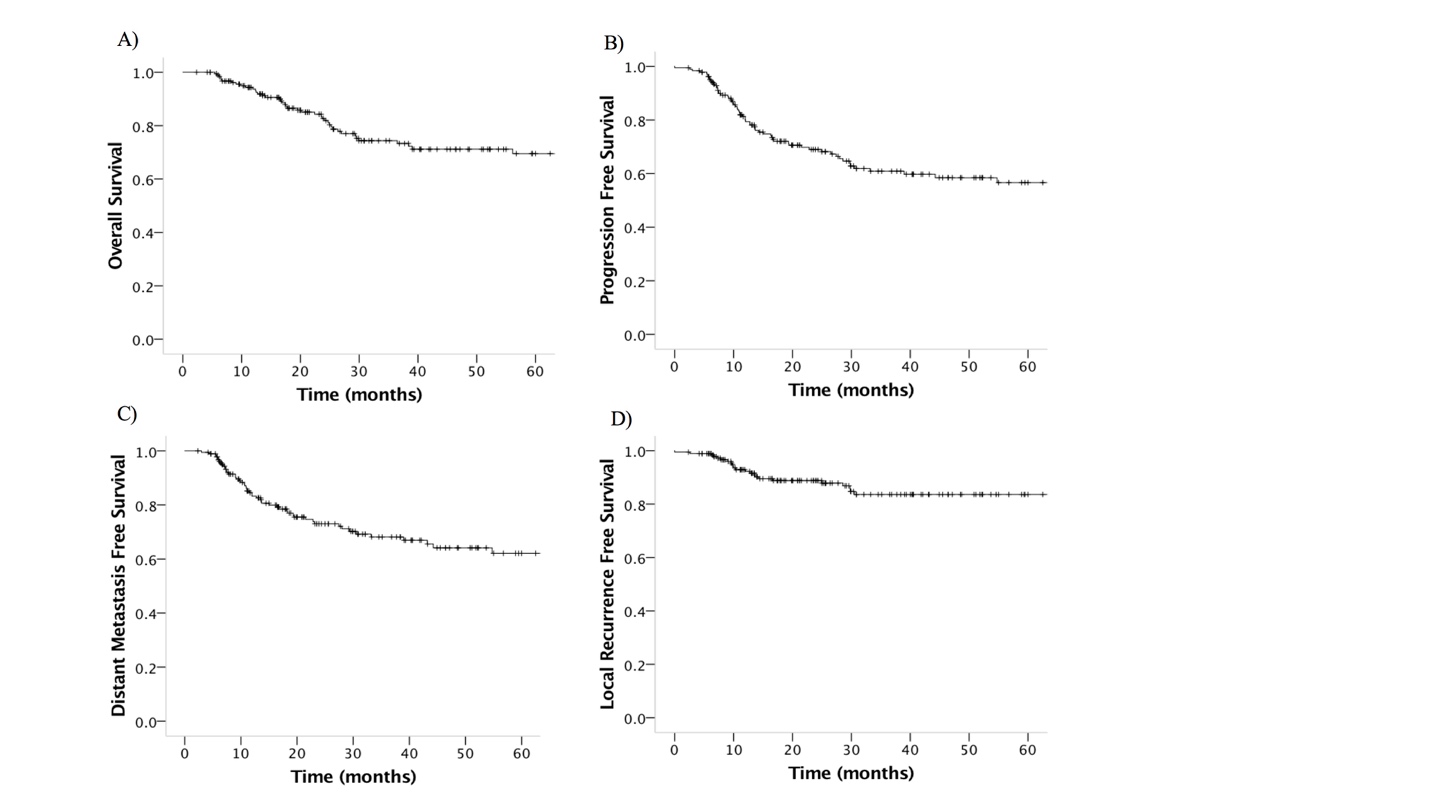
**

**Supplementary figure S1**

**
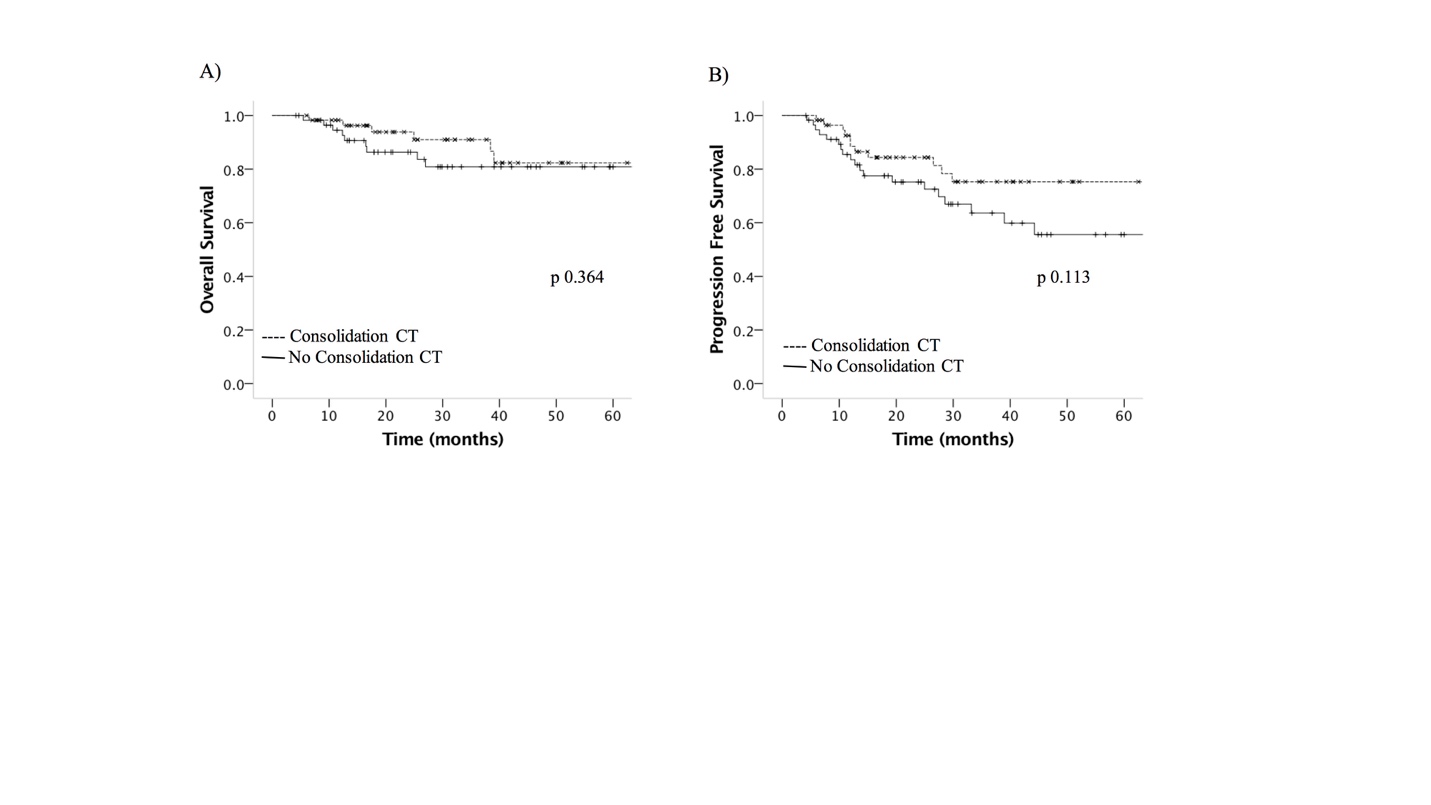
**

**Supplementary figure S2**


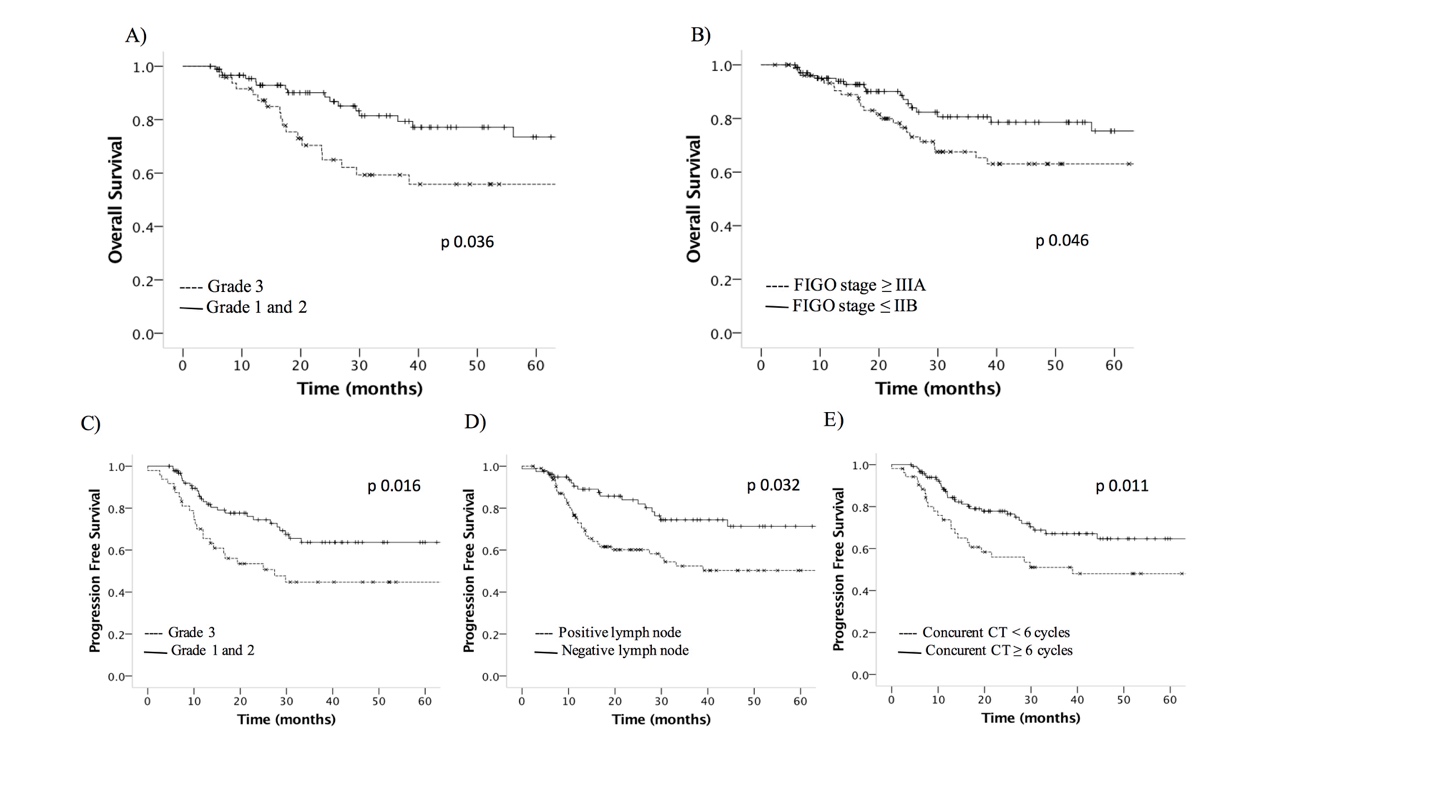


**Supplementary figure S3**


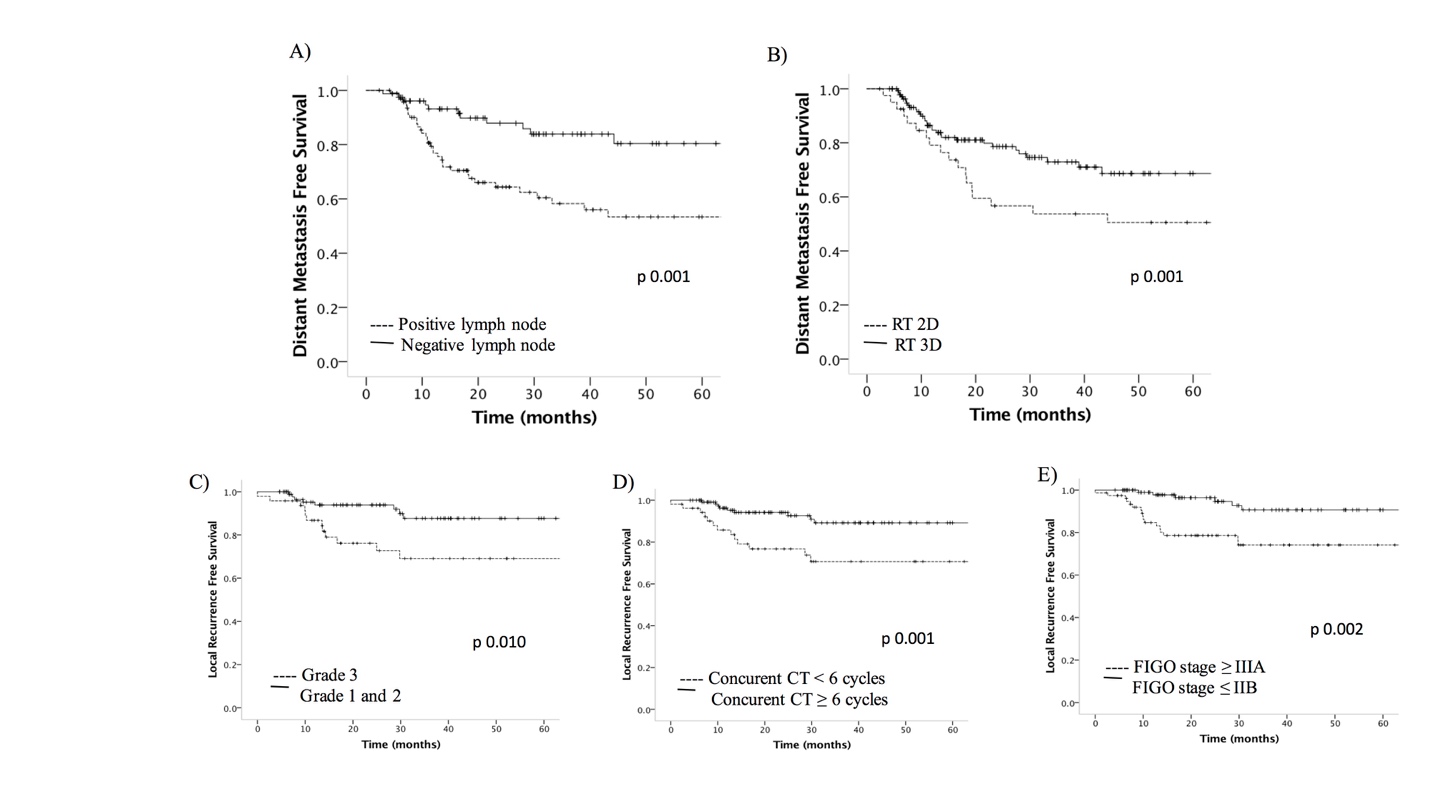


**Supplementary figure S4**

| **Supplementary table S1** | | | | |
| --- | --- | --- | --- | --- |
| **Characteristic** | **Freq. (%)** | |  | |
|  | **Consolidation CT** | **No Consolidation CT** | **p*** | |
| Number of patients | 58 | 58 |  | |
| Age (median/IQR) | 48.0 (41.0 – 61.0) | 47.5 (40.0 – 57.2) |  | |
| < 60 | 52 (89.7) | 48 (82.8) | 0.420 | |
| ≥ 60 | 6 (10.3) | 10 (17.2) |  | |
| ECOG performance status |  |  |  | |
| 0 | 36 (62.1) | 38 (65.5) | 0.847 | |
| ≥ 1 | 22 (37.9) | 20 (34.5) |  | |
| Histology |  |  |  | |
| Squamous cell carcinoma | 41 (70.7) | 41 (73.2) | 0.836 | |
| Adenocarcinoma | 17 (29.3) | 15 (26.8) |  | |
| Grade |  |  |  | |
| 1 and 2 | 34 (77.3) | 30 (63.8) | 0.177 | |
| 3 | 10 (22.7) | 17 (36.2) |  | |
| FIGO stage |  |  |  | |
| IB2 | 3 (5.2) | 6 (10.5) | 0.074 | |
| IIA2 | 2 (3.4) | 4 (7.0) |  | |
| IIB | 26 (44.8) | 32 (56.1) |  | |
| IIIA | 2 (3.4) | 3 (5.3) |  | |
| IIIB | 15 (25.9) | 9 (25.9) |  | |
| IVA | 9 (15.5) | 1 (1.8) |  | |
| IVB | 1 (1.7) | 2 (3.5) |  | |
|  |  |  |  | |
| Tumor size |  |  |  | |
| < 6cm | 28 (75.7) | 29 (80.6) | 0.778 | |
| ≥ 6cm | 9 (24.3) | 7 (19.4) |  | |
| Lymphnode |  |  |  | |
| Negative | 25 (43.1) | 28 (49.1) | 0.629 | |
| Pelvic | 32 (55.2) | 27 (47.4) |  | |
| Paraortic | 1 (1.7) | 2 (3.5) |  | |
| Concurrent CT < 6 cycles |  |  |  | |
| No | 46 (79.3) | 47 (81.0) | 1.000 | |
| Yes | 12 (20.7) | 11 (19.0) |  | |
| Radiotherapy technique |  |  |  | |
| 3D | 53 (91.4) | 54 (93.1) | 1.000 | |
| 2D | 5 (8.6) | 4 (6.9) |  | |
| *All p values calculated using Chi square test. CT = Chemotherapy. IQR = Interquartile Range. | | | |  |

| **Supplementary Table S2** | | | |
| --- | --- | --- | --- |
| **Characteristic** | **HR (95% CI)** | | **p value** |
| Age |  | |  |
| < 60 | 1 | | 0.277 |
| > 60 | 1.43 (0.75-2.72) | |  |
| ECOG performance status |  | |  |
| 0 | 1 | | 0.328 |
| > 1 | 1.36 (0.73-2.53) | |  |
| Histology |  | |  |
| Squamous cell carcinoma | 1 | | 0.329 |
| Adenocarcinoma | 1.43 (0.69-2.90) | |  |
| Grade |  | |  |
| 1 and 2 | 1 | | 0.040 |
| 3 | 1.99 (1.03-3.81) | |  |
| Tumor size |  | |  |
| < 6cm | 1 | | 0.709 |
| > 6 cm | 0.83 (0.30-2.22) | |  |
| FIGO Stage |  | |  |
| < IIB | 1 | | 0.050 |
| > IIIA | 1.83 (1.00-3.33) | |  |
| Lymph node |  | |  |
| negative | 1 | 0.220 | |
| positive | 1.49 (0.78-2.83) |  | |
| Concurrent CT < 6 cycles |  |  | |
| No | 1 | 0.062 | |
| Yes | 1.82 (0.97-3.42) |  | |
| Radiotherapy technique |  |  | |
| 3D | 1 | 0.056 | |
| 2D | 1.83 (0.98-3.39) |  | |
| Consolidation CT |  |  | |
| No | 1 | 0.021 | |
| Yes | 0.36 (0.15-0.85) |  | |

| CT = chemotherapy |
| --- |

| **Supplementary Table S3** | | | |
| --- | --- | --- | --- |
| **Characteristic** | **HR (95% CI)** | | **p value** |
| Age |  | |  |
| < 60 | 1 | | 0.462 |
| > 60 | 1.24 (0.70-2.18) | |  |
| ECOG performance status |  | |  |
| 0 | 1 | | 0.462 |
| > 1 | 1.22 (0.72-2.06) | |  |
| Histology |  | |  |
| Squamous cell carcinoma | 1 | | 0.110 |
| Adenocarcinoma | 1.58 (0.90-2.77) | |  |
| Grade |  | |  |
| 1 and 2 | 1 | | 0.018 |
| 3 | 1.95 (1.12-3.37) | |  |
| Tumor size |  | |  |
| < 6cm | 1 | | 0.966 |
| > 6 cm | 1.02 (0.45-2.26) | |  |
| FIGO Stage |  | |  |
| < IIB | 1 | | 0.065 |
| > IIIA | 1.60 (0.97-2.65) | |  |
| Lymph node |  | |  |
| negative | 1 | | 0.007 |
| positive | 2.16 (1.23-3.78) | |  |
| Concurrent CT < 6 cycles |  |  | |
| No | 1 | 0.012 | |
| Yes | 1.96 (1.15-3.32) |  | |
| Radiotherapy technique |  |  | |
| 3D | 1 | 0.091 | |
| 2D | 1.59 (0.92-2.73) |  | |
| Consolidation CT |  |  | |
| No | 1 | 0.006 | |
| Yes | 0.40 (0.21-0.77) |  | |
| CT = chemotherapy | | | |

| **Supplementary Table S4** | | | |
| --- | --- | --- | --- |
| **Characteristic** | **HR (95% CI)** | | **p value** |
| Age |  | |  |
| < 60 | 1 | | 0.340 |
| > 60 | 1.35 (0.73-2.48) | |  |
| ECOG performance status |  | |  |
| 0 | 1 | | 0.821 |
| > 1 | 0.93 (0.50-1.71) | |  |
| Histology |  | |  |
| Squamous cell carcinoma | 1 | | 0.117 |
| Adenocarcinoma | 1.65 (0.88-3.06) | |  |
| Grade |  | |  |
| 1 and 2 | 1 | | 0.133 |
| 3 | 1.60 (0.86-2.94) | |  |
| Tumor size |  | |  |
| < 6cm | 1 | | 0.605 |
| > 6 cm | 1.28 (0.50-3.24) | |  |
| FIGO Stage |  | |  |
| < IIB | 1 | | 0.228 |
| > IIIA | 1.41 (0.80-2.44) | |  |
| Lymph node |  | |  |
| Negative | 1 | | 0.002 |
| Positive | 2.81 (1.45-5.43) | |  |
| Concurrent CT < 6 cycles |  |  | |
| No | 1 | 0.064 | |
| Yes | 1.75 (0.96-3.17) |  | |
| Radiotherapy technique |  |  | |
| 3D | 1 | 0.024 | |
| 2D | 1.93 (1.08-3.43) |  | |
| Consolidation CT |  |  | |
| No | 1 | 0.013 | |
| Yes | 0.40 (0.19-0.82) |  | |
| CT = chemotherapy | | | |

| **Supplementary Table S5** | | | |
| --- | --- | --- | --- |
| **Characteristic** | **HR (95% CI)** | | **p value** |
| Age |  | |  |
| < 60 | 1 | | 1.000 |
| > 60 | 1.00 (0.37-2.69) | |  |
| ECOG performance status |  | |  |
| 0 | 1 | | 0.473 |
| > 1 | 1.36 (0.58-3.14) | |  |
| Histology |  | |  |
| Squamous cell carcinoma | 1 | | 0.168 |
| Adenocarcinoma | 1.88 (0.76-4.63) | |  |
| Grade |  | |  |
| 1 and 2 | 1 | | 0.015 |
| 3 | 3.05 (1.24-7.46) | |  |
| Tumor size |  | |  |
| < 6cm | 1 | | 0.960 |
| > 6 cm | 1.03 (0.28-3.75) | |  |
| FIGO Stage |  | |  |
| < IIB | 1 | | 0.003 |
| > IIIA | 4.02 (1.58-10.19) | |  |
| Lymph node |  | |  |
| Negative | 1 | | 0.142 |
| Positive | 1.96 (0.79-4.81) | |  |
| Concurrent CT < 6 cycles |  |  | |
| No | 1 | 0.004 | |
| Yes | 3.46 (1.48-8.10) |  | |
| Radiotherapy technique |  |  | |
| 3D | 1 | 0.853 | |
| 2D | 0.91 (0.33-2.46) |  | |
| Consolidation CT |  |  | |
| No | 1 | 0.054 | |
| Yes | 0.30 (0.09-1.02) |  | |
| CT = chemotherapy | | | |
